# POEMS syndrome: A case report

**DOI:** 10.1016/j.radcr.2026.05.084

**Published:** 2026-06-30

**Authors:** Maryam Masjedi Esfahani, Shamim Shafieioun, Hajar Zahedimehr

**Affiliations:** Radiology Department, School of Medicine, Isfahan University of Medical Sciences, Isfahan, Iran

**Keywords:** POEMS syndrome, Castleman disease, Neuropathy, Plasma cell neoplasm

## Abstract

POEMS syndrome is a rare multisystem disorder caused by a monoclonal plasma cell neoplasm, characterized by polyneuropathy, organomegaly, endocrinopathy, monoclonal plasma cell disorder, and skin changes. Due to its overlapping features with other neuropathies, diagnosis is often delayed, impacting prognosis. We report a 37-year-old male presenting with progressive limb weakness, inguinal lymphadenopathy, and spinal nerve root enhancement. Imaging revealed mixed lytic–sclerotic bony lesions, lymphadenopathies, hepatosplenomegaly, and spinal nerve root thickening and enhancement, which raised early suspicion for an underlying plasma cell dyscrasia rather than an isolated inflammatory neuropathy. Electrodiagnostic studies showed subacute demyelinating polyradiculoneuropathy with axonal degeneration, and laboratory findings included hypoalbuminemia, anemia, and thrombocytosis. A biopsy from the lymphadenopathy confirmed mixed plasma cell and hyaline vascular type Castleman disease, establishing POEMS syndrome in the context of a systemic plasma cell disorder. This case highlights how characteristic combinations of nerve root enhancement and mixed lytic–sclerotic bone lesions on MRI and CT can contribute uniquely to early diagnosis and differentiation from mimics such as chronic inflammatory demyelinating polyradiculoneuropathy or leptomeningeal disease, enabling prompt initiation of clone-directed therapy. Increased awareness of these imaging patterns among clinicians is critical for timely diagnosis and management.

## Introduction

POEMS syndrome is a rare, severe medical condition that is caused by a plasma cell neoplasm. The term POEMS stands for five things that are common to the disease: polyneuropathy, organomegaly, endocrinopathy, monoclonal plasma proliferative disorder, and skin abnormalities [1]. The disease is known as a rare plasma cell dyscrasia. It often shows up with additional critical clinical signs that aren't in the acronym, like sclerotic bone lesions, high levels of vascular endothelial growth factor (VEGF), papilledema, and too much fluid outside of blood vessels [2]. The neurological signature is usually a subacute onset demyelinating neuropathy that can quickly make patiant unable to move and cause discomfort. The plasma cell disease that causes it is almost always lambda confined [3].

The difficulties that physicians have in diagnosing POEMS syndrome, a rare disease, show how important it is to record individual instances. Because it is rare and looks like other neuropathies, like Chronic Inflammatory Demyelinating Polyneuropathy (CIDP), the condition is often not recognized or diagnosed correctly [4]. In reality, the diagnosis usually takes 12 to 16 months longer than it should, which makes people very incapacitated [5]. Because quick recognition and starting clone-directed therapy lead to the best results, a case report adds important clinical information to help frontline doctors who may be the first to see these different symptoms become more aware and make more accurate diagnoses.

Here, we report a 37-year-old male with progressive limb weakness due to demyelinating polyradiculoneuropathy and multi-focal lymphadenopathy and bony lesions attributed to a diagnosed mixed plasma cell and hyaline vascular type Castleman disease of the right inguinal lymph node.

## Case presentation

A 37-year-old male presented with progressive weakness in the lower limbs, which commenced two months before admission and later affected the upper limbs. He reported observing a distinct mass in the right inguinal region. On dermatologic examination, the only cutaneous abnormality identified was a newly developed erythematous papular lesion consistent with a red mole in the left axillary region, which had appeared about one month prior to admission.

Neurological imaging indicated a normal brain MRI, whereas the spinal MRI showed a lytic lesion in the L1 vertebral body extending into the left pedicle (Fig. 1). Moreover, there was notable thickening and contrast enhancement of the spinal nerve roots, especially in the cauda equina region and conus medullaris (Fig. 2). The chest CT findings were unremarkable. Abdominopelvic CT scan demonstrated multiple enhancing right inguinal lymphadenopathies and several lytic and sclerotic bony lesions (Fig. 3). It also has demonstrated hepatosplenomegaly (Fig. 4). Ultrasound examination of the right inguinal region revealed multiple lymph nodes in the right iliac chain and inguinal canal, exhibiting oval morphology, vascular stalks, and occasional mild hypoechogenicity. The maximum short-axis diameter (SAD) recorded was 30 mm. The ultrasound-guided biopsy of the inguinal lymphadenopathy confirmed the diagnosis of Castleman disease (mixed type). The immunohistochemical analysis of the lymph node biopsy demonstrated CD20 positivity in germinal centers, signifying B-cell proliferation. CD3 exhibited positivity in parafollicular lymphocytes, indicating the presence of T-cell zones. CD21 exhibited positivity in expanded follicular dendritic cells, aligning with the characteristics of follicular hyperplasia. CD5 negativity aids in the exclusion of specific T-cell lymphomas, while CD138 positivity in germinal centers indicates plasma cell infiltration.

**Fig. 1:**
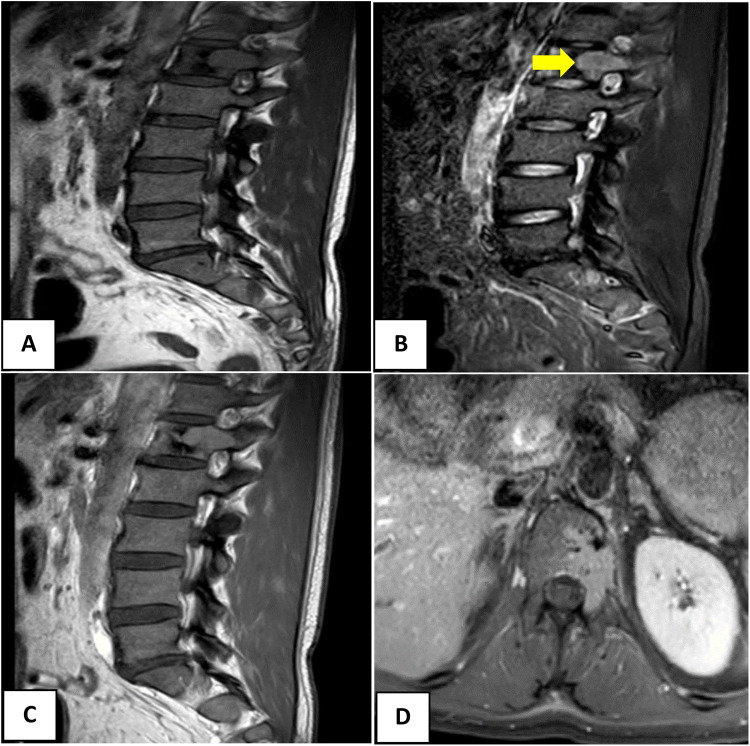
Sagittal and axial spinal MRI demonstrating an enhancing vertebral lesion. Sagittal T1-weighted image without contrast (A) and sagittal T2-weighted fast spin-echo image (B) show a lesion in the L1 vertebral body extending into the left pedicle. Sagittal (C) and axial (D) fat-suppressed T1-weighted post-contrast images after intravenous gadolinium administration demonstrate intense enhancement of the lesion (yellow arrow).

**Fig. 2:**
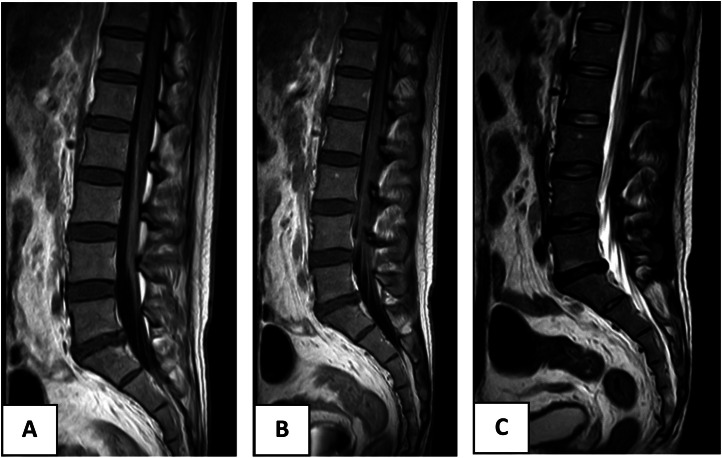
Spinal MRI showing cauda equina nerve root thickening and enhancement. Sagittal T1‑weighted post‑contrast images (A, B) and sagittal T2‑weighted fast spin‑echo image (C) demonstrate diffuse thickening, T2 hyperintensity, and contrast enhancement of the spinal nerve roots, particularly in the cauda equina and conus medullaris regions.

**Fig. 3:**
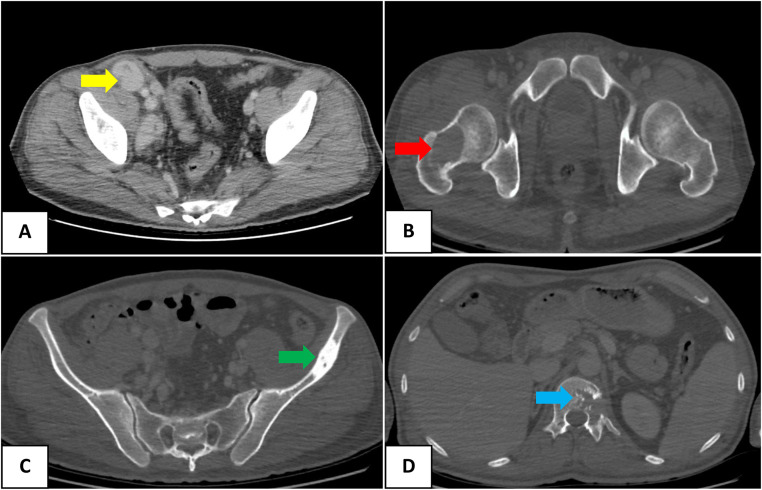
Abdominopelvic CT demonstrating lymphadenopathy and osseous lesions (axial views). Contrast-enhanced abdominopelvic CT in axial planes shows multiple enhancing right inguinal lymph nodes (A), a lytic bone lesion (B), a sclerotic bone lesion (C), and an additional lytic bone lesion (D).

**Fig. 4:**
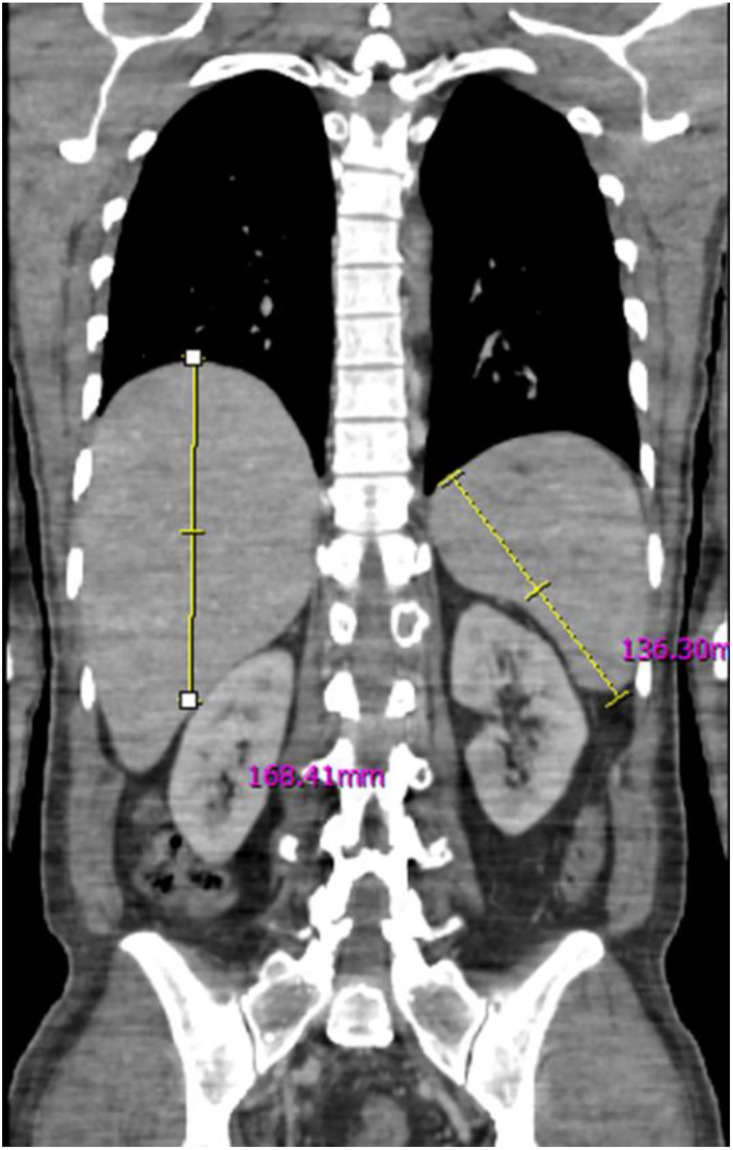
Abdominopelvic CT demonstrating organomegaly (coronal view). Contrast-enhanced abdominopelvic CT in the coronal plane reveals hepatosplenomegaly.

Electrodiagnostic studies, comprising electromyography (EMG) and nerve conduction studies (NCS), indicated subacute demyelinating polyradiculoneuropathy with significant secondary axonal degeneration.

Laboratory investigations indicated mild hypoalbuminemia (53.6 g/L), slightly below the normal range, and elevated alpha-1 and alpha-2 globulin fractions, which suggest an acute phase response or underlying inflammatory activity (Fig. 5). The complete blood count indicated mild anemia, characterized by a reduced red blood cell count of 4.16 M/µL, hemoglobin level of 13.6 g/dL, and hematocrit of 41.4%. The platelet count was elevated at 489,000 /µL, suggesting potential reactive thrombocytosis. The white blood cell count and differential fell within normal limits, exhibiting a balanced distribution of neutrophils, lymphocytes, and monocytes. The findings indicate a chronic inflammatory or reactive process. VEGF testing, which could have provided additional biochemical support for POEMS syndrome, was not performed because this assay was not routinely available in our laboratory.

**Fig. 5:**
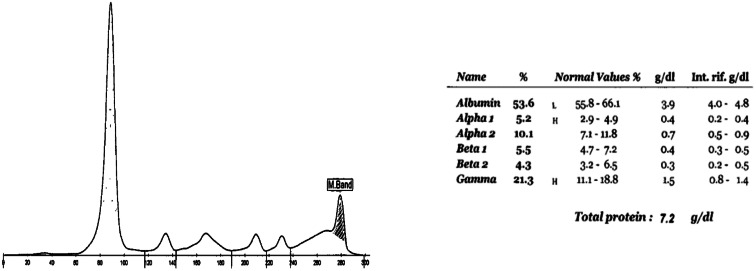
Serum protein capillary electrophoresis.

Following confirmation of POEMS syndrome associated with mixed-type Castleman disease, the patient was referred to a multidisciplinary team including neurology, hematology–oncology, and radiation oncology for clone-directed therapy and supportive care. Because of multifocal osteosclerotic and mixed lytic–sclerotic bone lesions, systemic therapy was favored over isolated radiotherapy. He received induction chemotherapy with lenalidomide (10 mg) on days 1-21 of a 28-day cycle plus weekly dexamethasone (days 1, 8, 15, and 22), one cycle every 28 days for a total of 12 cycles. Upon presentation, the patient demonstrated extremely weak extremities, a condition known as quadriplegia. In the upcoming follow-up visits, the patient has shown gradual, although not complete, neurological recovery along with partial restoration of motor function at the joints of both the upper and lower extremities, resulting in less severe residual quadriparesis.

## Discussion

The current diagnostic criteria for the POEMS syndrome in our patient include several factors which fall under the category of POEMS syndrome, a multisystemic disease that involves mandatory major criteria, other major criteria, and minor criteria. The diagnosis of the POEMS syndrome requires mandatory criteria of polyneuropathy and monoclonal plasma cell proliferation disorders; at least one more major criteria of sclerotic bone lesions, raised level of VEGF, or Castleman disease, and at least one minor criterion out of the list of organomegaly, endocrine disorder, dermatological changes, papilledema, extravascular volume overload, and thrombocytosis. From these criteria, skin-related problems can be considered to be minor criteria [1,6,7].

The patient demonstrated a gradual, ascending loss of strength starting in the lower limbs and subsequently involving the upper limbs, consistent with the polyneuropathy characteristic of POEMS syndrome. Electrodiagnostic studies confirmed the presence of subacute demyelination of polyradiculoneuropathy with subsequent axonal degeneration [8], a condition that confirms POEMS and can differentiate POEMS from other conditions such as chronic inflammatory demyelinating polyneuropathy (CIDP) [9]. Spinal MRI additionally showed diffuse, largely symmetric thickening and strong enhancement of the cauda equina and conus medullaris nerve roots without significant cerebrospinal fluid space effacement. This appearance is compatible with POEMS-related demyelinating polyradiculoneuropathy, in which smooth, symmetric nerve root enlargement and enhancement are typical. In our patient, the combination of smooth root enlargement on spinal MRI and mixed lytic–sclerotic vertebral and pelvic lesions on CT prompted early suspicion of a systemic plasma cell dyscrasia rather than primary inflammatory neuropathy.

By comparison, CIDP can show similar root hypertrophy and enhancement but usually lacks the associated osteosclerotic or mixed bone lesions, organomegaly, endocrinopathy, and monoclonal plasma cell disorder that characterize POEMS. Leptomeningeal disease more often produces nodular or irregular enhancement with greater cerebrospinal fluid space obliteration and is typically linked to an underlying systemic malignancy, while infectious radiculitis tends to be more focal or segmental and accompanied by meningeal involvement and cerebrospinal fluid inflammatory changes. In our patient, the combination of smooth root enlargement, preserved cerebrospinal fluid spaces, and the absence of clinical or laboratory evidence for neoplastic leptomeningeal or infectious processes supports the interpretation that the spinal findings reflect POEMS-related polyradiculoneuropathy rather than alternative etiologies.

Radiological assessments identified a lytic lesion at L1 along with multiple additional bony lesions, which are crucial for satisfying the additional major diagnostic criteria. Bony lesions in POEMS can be heterogeneous, often including osteosclerotic or mixed lytic–sclerotic forms, and MRI commonly shows thickening and enhancement of nerve roots along with other characteristic features [10]. In our patient, recognizing this combination of mixed osseous lesions and demyelinating polyradiculoneuropathy on imaging was pivotal in distinguishing POEMS syndrome from isolated inflammatory neuropathies and from purely metastatic involvement, and it guided subsequent targeted histopathological evaluation of the inguinal lymphadenopathy.

From a therapeutic standpoint, POEMS syndrome is managed by targeting the underlying monoclonal plasma cell clone, with the choice of modality guided by the pattern and extent of bone and marrow involvement. Patients with a solitary or limited osteosclerotic lesion may achieve durable control with involved-field radiotherapy alone, whereas those with multifocal sclerotic or mixed lytic–sclerotic lesions or diffuse marrow disease, as in our patient, generally require systemic myeloma-directed therapy using alkylators and corticosteroids, immunomodulatory drugs such as lenalidomide, and when eligible, high-dose melphalan followed by autologous stem cell transplantation [11]. Contemporary series and systematic reviews indicate that these clone-directed regimens can induce substantial hematological and neurological responses and have markedly improved long-term survival in POEMS syndrome [12].

In our imaging work-up, contrast-enhanced CT of the chest, abdomen, and pelvis together with spinal MRI was performed, which did not reveal additional large-volume lesions beyond those described; however, whole-body low-dose CT or positron emission tomography-computed tomography (PET/CT) was not undertaken, and thus the presence of small or metabolically less conspicuous lesions elsewhere cannot be entirely excluded. Nevertheless, the available CT and MRI studies were sufficient to demonstrate the characteristic constellation of nerve root enhancement, mixed lytic–sclerotic bone lesions, lymphadenopathy, and organomegaly, thereby compensating in part for the lack of serum VEGF measurements and supporting an early diagnosis of POEMS syndrome. Taken together, the spinal MRI and systemic CT findings in this patient illustrate an imaging pattern that should raise early suspicion for POEMS syndrome: smooth, symmetric cauda equina nerve-root thickening and enhancement; multifocal mixed lytic–sclerotic bone lesions; right inguinal lymphadenopathy; and hepatosplenomegaly. Recognizing this constellation as a unifying process, rather than as separate spinal, osseous, and nodal abnormalities, can prompt timely investigation for an underlying plasma cell dyscrasia and avoid anchoring on more common isolated neuropathies or metastatic disease.

One of the distinguishing characteristics seen in this case is that of Castleman disease, which was also diagnosed through histopathological analysis, and further supports the diagnosis of POEMS syndrome. Castleman disease affects some POEMS cases, and of the types, it is the hyaline vascular type that is commonly associated [1]. Laboratory tests indicated mild hypoalbuminemia, elevated globulin fractions, mild anemia, and thrombocytosis. The elevated platelet count is a recognized minor criterion associated with POEMS syndrome, while mild anemia and hypoalbuminemia provide additional context for the patient's overall health and prognosis, as low serum albumin is a critical prognostic indicator [13]. The confirmation of Castleman disease along with patterns of B-cell proliferation indicates the presence of a monoclonal plasma cell disorder, fulfilling essential diagnostic criteria for POEMS syndrome [1].

From a diagnostic point of view, this case is a typical example of a common error when the initial diagnosis of POEMS syndrome is CIDP or other acquired demyelinating neuropathy. In patients with subacute demyelinating polyradiculoneuropathy, the recognition of POEMS and the initiation of clone-directed therapy may be delayed due to the failure to actively search for mixed lytic–sclerotic bone lesions, organomegaly, endocrinopathy, thrombocytosis, and a monoclonal plasma cell disorder. Consequently, it is imperative to conduct a systematic evaluation of these systemic features whenever CIDP or related neuropathies are taken into account in the differential diagnosis.

Overall, the complexity of the current case illustrates the association among demographic, clinical presentation, and laboratory studies needed for diagnosis of POEMS syndrome and the pathological understanding of the disease. The cumulative clinical information and selective spinal MRI along with systemic CT scans illustrate the necessity of using a comprehensive method for diagnosis of this entity. It is also important to note that the current case illustrates the ability to identify POEMS syndrome using a specific pattern of nerve-root enhancement, lytic and sclerotic bony lesions, and organomegaly and lymphadenopathy, aiding in differentiating it from other more common inflammatory or neoplastic diseases.

## Conclusions

This case report thoroughly demonstrates a validated diagnosis of POEMS syndrome. Our patient fulfilled the mandatory criteria of polyneuropathy, evidenced by a subacute demyelinating polyradiculoneuropathy with subsequent axonal degeneration, and a monoclonal plasma cell proliferative disorder, corroborated by the co-occurrence of Castleman disease (mixed type). In addition, the patient exhibited a major criterion, namely multiple lytic and sclerotic osseous lesions, and a minor criterion, thrombocytosis, which is a recognized supportive feature of POEMS syndrome. This case emphasizes the imperative for increased clinical surveillance and prompt intervention aimed at the underlying clonal plasma cell dyscrasia to attain sustained remission and enhance long-term survival.

## Patient consent

The written, informed consent for publication of their case was obtained from the patient.
